# Generating Multidirectional Variable Hidden Attractors via Newly Commensurate and Incommensurate Non-Equilibrium Fractional-Order Chaotic Systems

**DOI:** 10.3390/e23030261

**Published:** 2021-02-24

**Authors:** Nadjette Debbouche, Shaher Momani, Adel Ouannas, ’Mohd Taib’ Shatnawi, Giuseppe Grassi, Zohir Dibi, Iqbal M. Batiha

**Affiliations:** 1 Department of Mathematics and Computer Science, University of Larbi Ben M’hidi, Oum El Bouaghi 04000, Algeria; nadjette.debbouche@gmail.com (N.D.); ouannas.adel@univ-oeb.dz (A.O.); 2 Department of Mathematics, Faculty of Science, The University of Jordan, Amman 11942, Jordan; s.momani@ajman.ac.ae; 3 Nonlinear Dynamics Research Center (NDRC), Ajman University, Ajman 346, United Arab Emirates; 4 Department of Basic Science, Al-Huson University College, Al-Balqa Applied University, Irbid 21510, Jordan; taib.Shatnawi@bau.edu.jo; 5 Dipartimento Ingegneria Innovazione, Universita del Salento, 73100 Lecce, Italy; giuseppe.grassi@unisalento.it; 6 Department of Electronics, University of Batna 2, Batna 05000, Algeria; zohirdibi@yahoo.fr; 7 Department of Mathematics, Faculty of Science and Technology, Irbid National University, Irbid 2600, Jordan

**Keywords:** Caputo fractional-order operator, commensurate and incommensurate fractional-order derivative, hidden attractors, dynamic states, bursting, inversion property, coexisting attractors, offset boosting

## Abstract

This article investigates a non-equilibrium chaotic system in view of commensurate and incommensurate fractional orders and with only one signum function. By varying some values of the fractional-order derivative together with some parameter values of the proposed system, different dynamical behaviors of the system are explored and discussed via several numerical simulations. This system displays complex hidden dynamics such as inversion property, chaotic bursting oscillation, multistabilty, and coexisting attractors. Besides, by means of adapting certain controlled constants, it is shown that this system possesses a three-variable offset boosting system. In conformity with the performed simulations, it also turns out that the resultant hidden attractors can be distributively ordered in a grid of three dimensions, a lattice of two dimensions, a line of one dimension, and even arbitrariness in the phase space. Through considering the Caputo fractional-order operator in all performed simulations, phase portraits in two- and three-dimensional projections, Lyapunov exponents, and the bifurcation diagrams are numerically reported in this work as beneficial exit results.

## 1. Introduction

The dynamical system of fractional order is certainly deemed as a generalization structure of the Integer-order System (IoS) [1,2]. Such system in its fractional-order case has been employed in a broad spectrum of applied sciences such as materials engineering, general mechanics, electrical circuit, physics, etc. [3,4,5,6,7]. Recently, it has been shown that Fractional-order Differential Equations (FoDEs) can be much better than Ordinary Differential Equations (ODEs) for describing many physical phenomena [8]. For this reason, many scholars have been progressively motivated to deeply explore the Fractional-order Systems (FoSs) in their chaotic modes. Until now, several chaotic FoSs have been broadly analyzed, in particular regarding the fractional-order Lorenz system [2,9], the fractional-order Chua system [10], and the fractional-order Chen system [11].

More recently, lots of efforts have been devoted to the FoSs that have no equilibrium points, generating complex chaotic behaviors for their modes [12,13,14]. In particular, these systems can exhibit special attractors named hidden attractors. In fact, there are two classes of chaotic attractors: the so-called self-excited attractors and the hidden ones. It turns out that unstable equilibrium points do not have a limited neighborhood in which they connect with attraction basins of such attractors. This is absolutely different from the second class, in which an unstable equilibrium can excite it. In general, the nonlinear system that has either stable- or line-equilibrium points, or even none of them, can exhibit such hidden attractors. Due to the absence of any equilibrium, it is extremely complicated to numerically place the attractors of the FoSs through employing the Standard Computational Procedure (SCP), in contrast with a self-excited attractor that could be identified via the SCP itself. The hidden attractors could be considered an exceedingly critical problem, especially in some engineering applied subjects. This, however, returns to their abilities in generating some disastrous perturbations and other unexpected responses to, e.g., the infrastructure of a bridge or even the body of an aircraft wing [15,16,17].

In other respects, as a consequence of the resultant chaotic signals from the chaotic system with variable attractors that can be designed with any polarity, such system is deemed as an appropriate solution for many chaos-based applied studies in which it can diminish the electronic components needed for signal conditioning. In other words, the position of the chaotic attractor will definitely be variable in the phase space, and it could be arbitrarily selected in accordance with the parameters of the offset control. In recent years, diverse literature has addressed various chaotic FoSs with one- and two-boostable variables [8,18,19,20,21,22,23]. Only a few works have addressed these systems with three-boostable variables [20,24].

In [25], Zhang et al. established a novel non-equilibrium chaotic IoS of three dimensions. This system is considered the most uncomplicated system in comparison with the other proposed non-equilibrium chaotic systems since it has a constant, a non-quadratic signum function, and a straightforward linear algebraic construction. Besides, this system holds three inconstant variables, and the hidden attractor is diffused along each line *x*, *y*, and *z*; inside each xz-, yz-, and xz-lattice; and within xyz-grid by inserting another three additional controlled constants. In addition, by using traditional nonlinear analysis schemes, some rich and complex hidden dynamic modes, e.g., the transient transition mode and the chaotic bursting mode, have been exposed and investigated numerically.

In view of the aforementioned considerations, this work intends to construct a new FoS based on the chaotic IoS that was recently proposed by Zhang et al. [25]. Besides, it intends to examine the impact of the incommensurate and commensurate fractional-order derivative on the FoS numerically. Different complex dynamical behaviors of the proposed commensurate and incommensurate FoS are explored and discussed through performing several numerical simulations. Such results are reported with the help of the phase portraits in 2D projections, Lyapunov Exponents (LEs), and bifurcation diagrams. The proposed FoS can exhibit different striking phenomena including inversion property, hidden bursting oscillation, and coexisting multiple attractors. This system can also be degenerated into a 1D line, 2D lattice, and 3D grid of variable hidden attractors by including offset boosting parameters for the fractional order in both commensurate and incommensurate cases.

The remaining part of this article is structured in the following order. In the next part, starting from the non-equilibrium IoS, the non-equilibrium FoS is modeled mathematically. In Section 3, several complicated and attractive dynamics of commensurate FoS are investigated in detail. Section 4 deals with such dynamics in accordance with the incommensurate case. The multidirectional variable hidden attractors generated from commensurate and incommensurate FoS are presented in Section 5. Section 6 summarizes the whole work.

## 2. A Non-Equilibrium FoS

A new 3D chaotic IoS was recently studied by [25]. This system can be expressed by three nonlinear DEs:

(1) $$\left\{\begin{array}{c} \dot{x}=\alpha sgn\left(y\right)+\beta z,\\ \dot{y}=\lambda +z,\\ \dot{z}=-\gamma x-z, \end{array}\right.$$

where α, β, and λ are nonnegative parameters, γ≠0 is constant, and sgn(y) represents the signum function that can be outlined as:

(2) $$sgn\left(y\right)=\left\{\begin{array}{c} 1,\;y>0\\ 0,\;y=0\\ -1,\;y<0. \end{array}\right.$$

**Figure 1:**
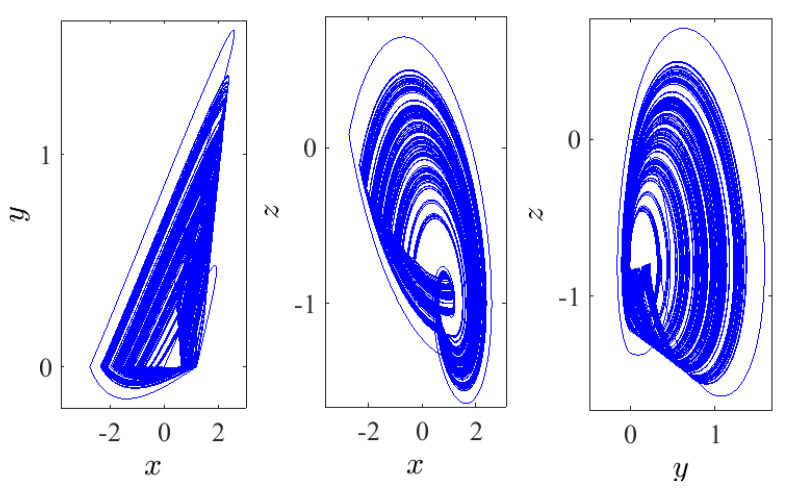
System (1) with its chaotic hidden attractors exhibited in distinct planes according to the IC (0,0,0) when α=2.8, β=2.8, γ=1, and λ=0.8.

In view of some selections of appropriate values for the system’s parameters together with the function sgn(y), as addressed in [25], system (1) has no equilibria, and a chaotic hidden attractor is exhibited according to the Initial Condition (IC) (0,0,0) for α=2.8, β=2.8, γ=1, and λ=0.8, as shown in Figure 1. However, we next state certain key preliminaries associated with the non-integer calculus [26]:

**Definition** **1.** *The integral operator of fractional-order q in the sense of Riemann–Liouville of the function $g\in C^{m}\left(0,T\right]$ is outlined as:*

(3) $$I^{q}g\left(t\right)=\frac{1}{\Gamma (q)}\int_{0}^{t}\frac{g(s)}{{\left(t-s\right)}^{\left(1-q\right)}}ds,$$

*where q>0, m∈N and T>0.*

**Definition** **2.** *The differential operator of fractional-order q in the sense of Caputo of the function $g\in C^{m}\left(0,T\right]$ is outlined as:*

(4) $$D^{q}g\left(t\right)=\left\{\begin{array}{c} \frac{1}{\Gamma (m-q)}\int_{0}^{t}{\left(t-s\right)}^{m-q-1}g^{\left(m\right)}\left(s\right)ds,\;q\in \left(m-1,m\right),\\ g^{\left(m\right)}\left(t\right),\;\;\;\;\;\;q=m, \end{array}\right.$$

*where q∈[m−1,m], m∈N, and T>0.*

From now on, we intend to generalize the IoS reported in (1) by considering the following FoD:

(5) $$\left\{\begin{array}{c} D^{q_{1}}x=\alpha sgn\left(y\right)+\beta z,\\ D^{q_{2}}y=\lambda +z,\\ D^{q_{3}}z=-\gamma x-z, \end{array}\right.$$

where $D^{q_{j}}$ is the Caputo’s operator of order $0<q_{j}\leq 1$, j=1,2,3, and *x*, *y*, and *z* are the system’s variables. Observe that this system is of commensurate order if $q_{1}=q_{2}=q_{3}$, otherwise it is called an incommensurate system. In this work, although the same parameter values of system (5) are taken as in [25], this system has no equilibrium point. For all performed numerical simulations, the so-called Adams–Bashforth–Moulton Predictor–Corrector (ABMPC) method [27,28] is extensively employed to study all resultant behaviors.

The Caputo operator is considered an extremely useful operator in modeling phenomena which take into account the interactions in the past as well as problems with nonlocal properties. From this perspective, the ABMPC method and the Benettin–Wolf (BW) algorithm employ such operator in their constructions. Actually, this is the main reason that leads us to use this operator in this work. For real-world engineering applications, a simple and reliable hardware electronic circuit for generating hidden chaotic signals is a necessity. It is, therefore, of great importance to search for a no-equilibrium low-dimensional chaotic system having a very simple algebraic structure and circuit topology. This work, however, attempts to offer these properties by recalling the IoS (1) and proposing the FoS (5) in two cases: commensurate and incommensurate orders.

## 3. The Commensurate FoS

In this part, we intend to investigate different dynamics features of the commensurate FoS given in (5), including the dynamic states analysis of such system versus slight changes in the fractional-order values as well as some other slight changes in the values of system’s parameters, inversion property, bursting of hidden attractor, and coexisting hidden attractors.

### 3.1. Chaos vs. the Variety in the Fractional-Order Values

**Figure 2:**
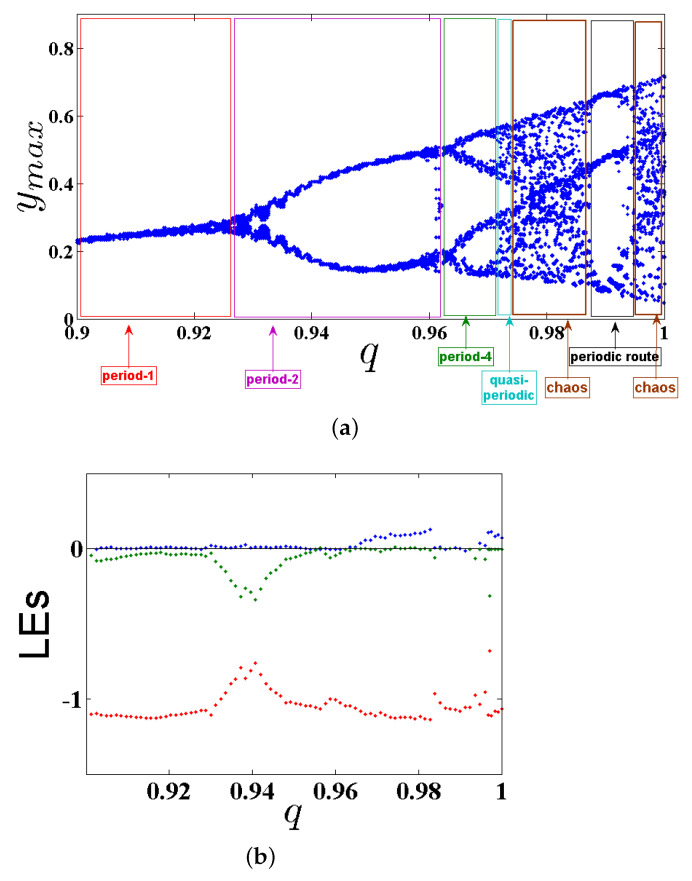
(**a**) The bifurcation diagram; and (**b**) LEs of system (5) with commensurate order by varying q∈(0.90,1) according to the IC (0,0,0), and the system’s parameters α=2.8, β=2.8, γ=1, λ=0.8.

The dynamic states analysis of system (5) in its commensurate order case is studied in this subsection through varying the fractional-order value *q* and fixing the at IC (0,0,0) together with the system’s parameters as: α=2.8, β=2.8, γ=1, and λ=0.8. In particular, one can see the bifurcation diagram when q∈(0.90,1) in Figure 2a. Based on this figure, different dynamic states of system (5) are presented in Table 1. One can observe that system (5) starts its evolution from Period 1, it is developed in Period 2, in Period 4 further changes are performed into the quasiperiodic state, and finally it is dropped into chaos when commensurate order q=0.9747. That is, the chaos exists when q∈[0.9747,0.988)∪[0.995,1].

**Figure 3:**
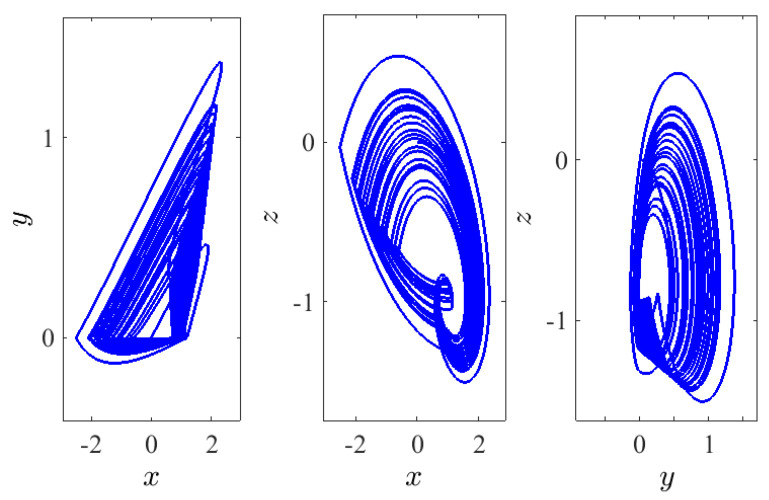
Chaotic hidden attractor of system (5) with commensurate order q=0.9747, shown on different planes according to the IC (0,0,0), and the system’s parameters α=2.8, β=2.8, γ=1, λ=0.8.

**Figure 4:**
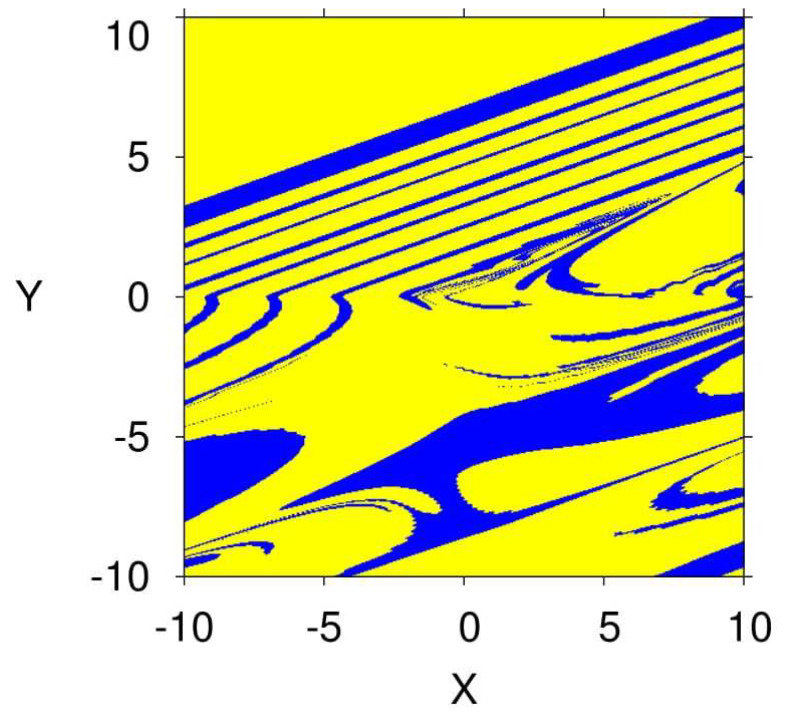
Basin of attraction section x−y of attractors shown in Figure 3 for q=0.98 according to the initial condition of the third state variable z=0.

At the same time, estimating the LEs is considered another numerical method employed for indicating chaos in the FoS given in (5), in which the existence of a chaotic behavior for such system can be indicated by the existence of positive LEs. Here, the Lyapunov exponents are denoted by $LE_{i},i=1,2,3$ with $LE_{1}>LE_{2}>LE_{3}$. Obviously, the proposed system is chaotic according to the values of the exponents bounded as $LE_{1}>0$, $LE_{2}=0$ and $LE_{3}<0$ with $|LE_{1}|<|LE_{2}|$. In [29], the BW algorithm is presented to identify all LEs for a category of FoSs established using the Caputo operator. Actually, this method cannot be implemented here because system (5) is classified as nonsmooth. Therefore, to calculate the LEs, we first use the same scheme presented in [30] considering the following substitution [31]:

sgn(y)→tanh(ρy),

where ρ is constant. In fact, this smooth approximation of the signum function allows estimating the LEs using the BWA. Indeed, using this algorithm has helped us to calculate all LEs of system (5) which can be seen in Figure 2b for ρ=15. For the fractional order q=0.9747, the three LEs are $LE_{1}=0.08,LE_{2}=0,LE_{3}=-1.29$ and |0.08|<|−1.29|. The fractional dimension, which presents the complexity of attractor, is defined by:

$$D_{KY}=j+\frac{1}{LE_{j+1}}\sum_{i=1}^{j}LE_{i},$$

where *j* is the largest integer satisfying $\sum_{i=1}^{j}LE_{i}\geq 0$ and $\sum_{i=1}^{j+1}LE_{i}<0$. The calculated dimension of system (5) when q=0.9747 is $D_{KY}=2.0620>2$. Consequently, a chaotic attractor is detectable in the system (see Figure 3). Besides, as a result of system (5) having no equilibria, the detecting chaotic attractor is hidden with one scroll, as shown in Figure 3 on different planes according to q=0.9747, the IC (0,0,0), and the system’s parameters α=2.8, β=2.8, γ=1, and λ=0.8. Figure 4 presents the basin of attraction of system (5) for q=0.98. In this figure, we observe that the ICs represented by the yellow region lead to unbounded orbits, whereas the other ICs represented by the blue region lead to a chaotic attractor. Besides, we should note that there is no fixed point in the considered system for the selected parameters. This implies that such chaotic attractor is hidden.

### 3.2. Chaos vs. the Variety in the Values of System’s Parameters

**Figure 5:**
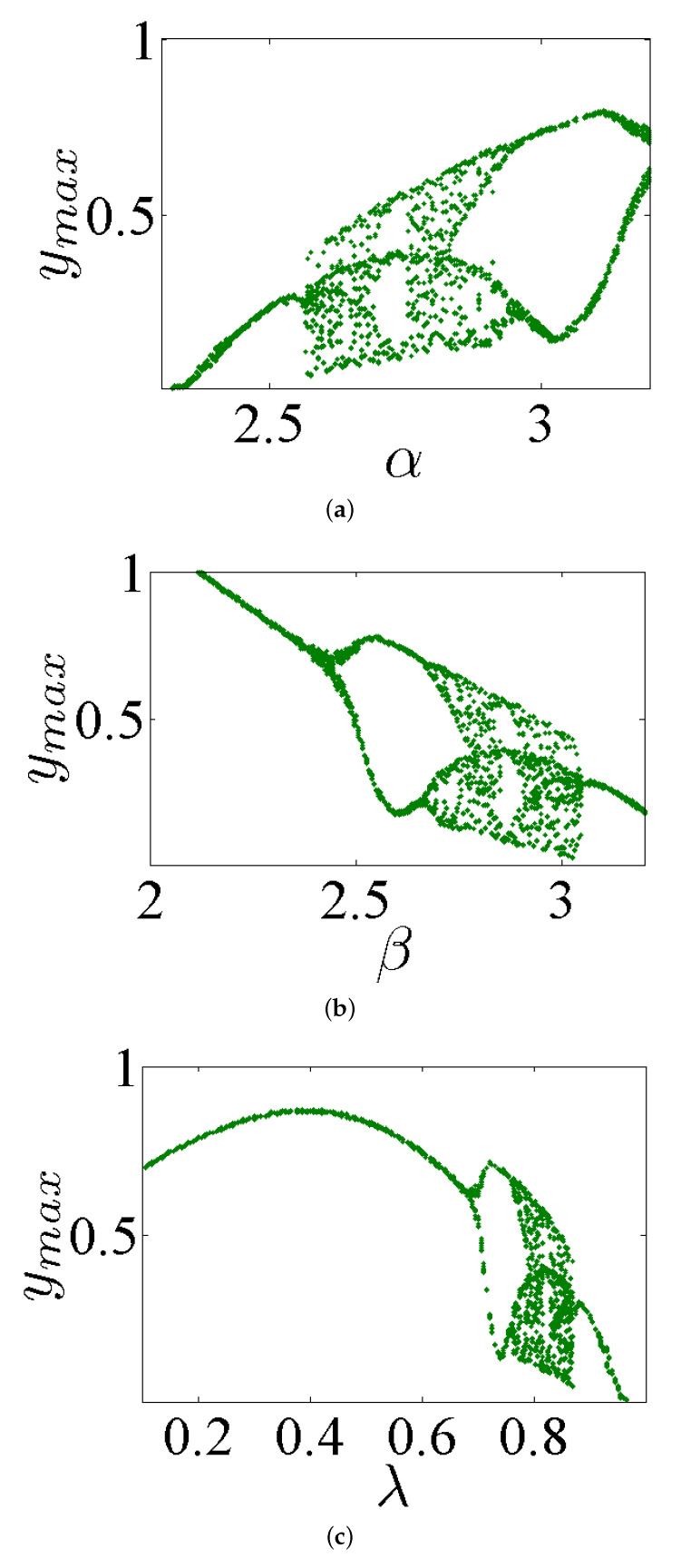
Bifurcation diagrams of system (5) with commensurate q=0.98 through fixing γ=1 and varying the parameters (**a**) α∈(2.3,3.2), (**b**) β∈(2,3.2), and (**c**) λ∈(0.1,1) according to the IC (0,0,0).

In this part, a bifurcation analysis of system (5) with its commensurate order q=0.98 is discussed by varying the system’s parameters α, β, and λ, while fixing the parameter γ=1. In accordance with the IC (0,0,0), several bifurcation diagrams of system (5) are demonstrated in Figure 5. In particular, for α∈(2.3,3.2), the bifurcation diagram is shown in Figure 5a. Based on this figure, it can be observed that, as α is reduced, system (5) displays a periodic route of Period 2 and Period 4. Besides, such system is then turned from a quasiperiodic state to chaos when α=2.85. In general, this chaotic behavior still exists until α=2.75. After this value, the system appears again in a periodic state, while it appears in a chaotic state from α=2.58 to α=2.68. In addition, this chaotic behavior disappears after α=2.58.

Figure 5b demonstrates the bifurcation diagram for β∈(2,3.2). Observe that, once the parameter β is increased, system (5) appears in a periodic route of Periods 1–2–4, and then it turns from a quasiperiodic state to a chaos state when β=2.79. This chaotic behavior still exists until β=2.85. After this value, a periodic state again appears for this system, and then it is in a chaotic state from β=2.91 to β=3.04. Afterward, at β=3.04, the chaotic behavior of this system disappears.

Finally, Figure 5c shows the bifurcation diagram for λ∈(0.1,1). It can be noted that system (5) turns from a periodic route to a chaos state when λ=0.79. Such chaotic behavior still exists as λ∈(0.79,0.81)∪(0.83,0.87), and the overall chaos state disappears after this range.

### 3.3. Inversion Property

**Figure 6:**
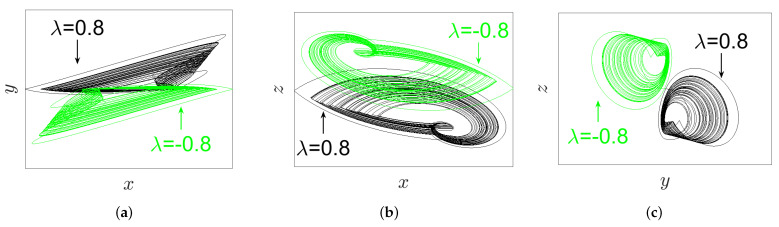
The phase portraits of system (5) with its commensurate order q=0.98 according to IC (0,0,0), and the parameters’ values α=2.8, β=2.8, γ=1, λ=±0.8 on different projections (black plot for λ=0.8, green plot for λ=−0.8): (**a**) xy-plane; (**b**) xz-plane; and (**c**) yz-plane.

**Figure 7:**
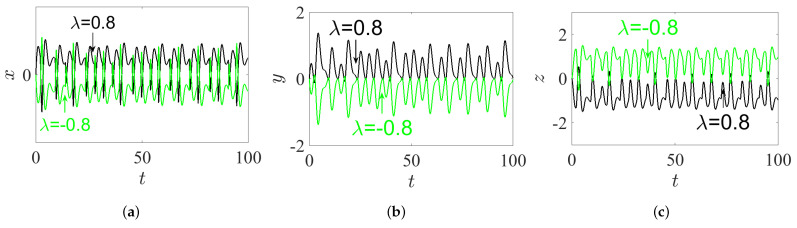
The time series of system (5) with its commensurate order q=0.98, corresponding to its IC (0,0,0) and its parameters α=2.8, β=2.8, γ=1, and λ=±0.8 (black plot for λ=0.8, green plot for λ=−0.8): (**a**) the state-space variable *x*; (**b**) the state-space variable *y*; and (**c**) the state-space variable *z*.

In [25], Zhang et al. reported that, for all signals (*x*, *y*, and *z*) of the IoS given in (1), the parameter λ possesses an inversion control. This means that the polarity of these signals is altered when the polarity of the parameter λ is changed. In this subsection, we find that it is interesting to explore whether this property still exists or not for the proposed FoS. For this reason, and according to the IC (0,0,0), we take q=0.98, α=2.8, β=2.8, and γ=1 to plot the phase portraits on different projections as well as the time series graph of system (5) for two opposite values of parameter λ=±0.8, as demonstrated, respectively, in Figure 6 and Figure 7. In view of these two figures, it can be pointed out that, when the polarity of term λ is changed, the polarity of all signals *x*, *y*, and *z* are also changed. In other words, the inversion property still exists in the FoS.

### 3.4. Hidden Bursting Oscillation

**Figure 8:**
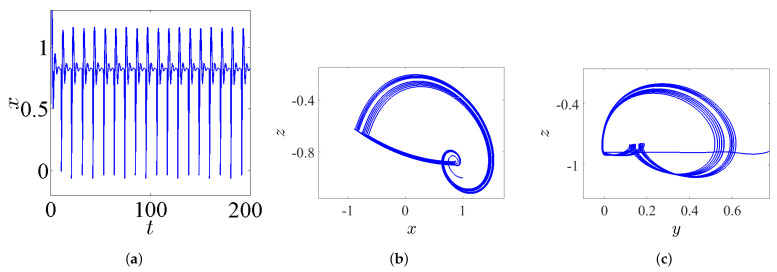
The chaotic bursting oscillation according to the IC (0,0,0), with q=0.985, α=2.8, β=3.4, γ=1, and λ=0.8: (**a**) the time series of the state-space variable *x*; (**b**) the phase portrait in xz-plane; and (**c**) the phase portrait in yz-plane.

**Figure 9:**
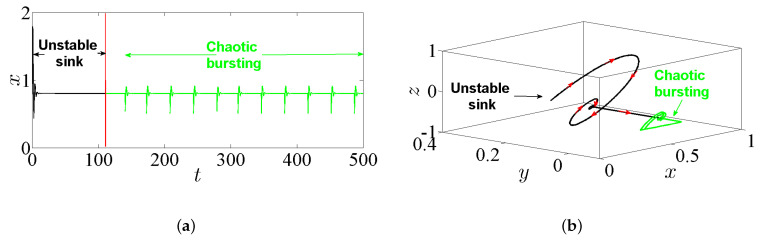
Passing transition behavior according to the IC (1,1,−1), with q=0.985, α=2.8, β=3.2, γ=1, and λ=0.8: (**a**) the corresponding time series of the state-space variable *x*; and (**b**) the phase portrait in 3D projection.

The bursting is a particular complex nonlinear practical application which can be witnessed as a significant communication operation in, e.g., endocrine cells and biological neurons [32]. In general, the bursting arises due to the trajectory that subdues several transitions between the fast subsystem’s attractors. These transitions can be adapted by the sluggish variable once it periodically crosses through the fast subsystem’s bifurcation points [33]. Actually, this exciting application has been extensively handled in several nonlinear FoSs [34,35]. However, the time series of the state-space variable *x* together with the phase portraits of system (5) are plotted in Figure 8 by taking α=2.8, β=3.4, γ=1, λ=0.8, and q=0.985 and assuming the IC (0,0,0). For instance, the time series in Figure 8a shows a periodic bursting oscillations, whereas t Figure 8b,c shows phase portraits which exhibit the chaotic bursting pattern. In particular, when one chooses q=0.985, α=2.8, β=3.2, γ=1, λ=0.8, and the IC as (1,1,−1), a new kind of behavior associated with passing transition of system (5) is noticed clearly. For more insight, Figure 9a shows the time-domain waveform of the state-space variable *x*, while Figure 9b shows its corresponding phase portrait in 3D projection. It can be remarked from these two figures that the trajectories of the FoS given in (5) incur a transition that begins at an unstable sink and ends at a steady chaotic bursting oscillation with the evolution of time, resulting in a complex behavior of the state transition.

### 3.5. Coexisting Hidden Attractors

**Figure 10:**
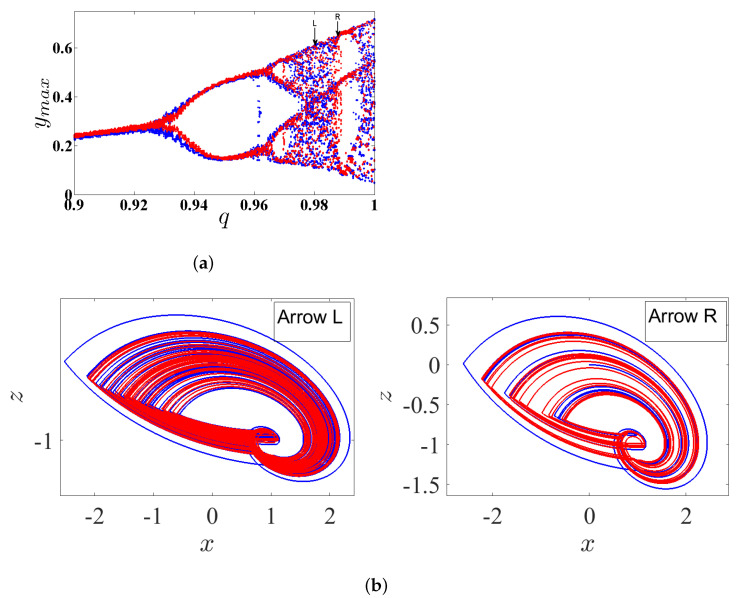

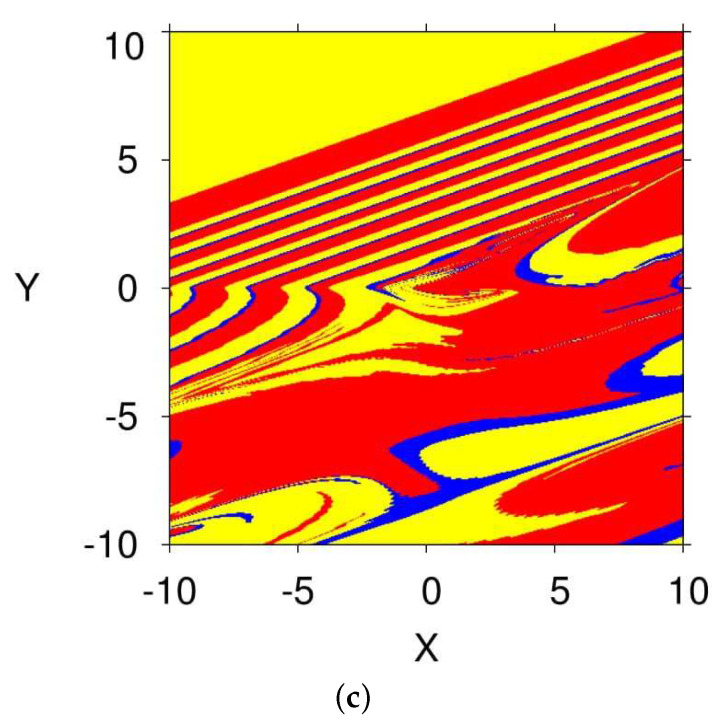
(**a**) The diagram of bifurcation of the FoS given in (5) for *q* ∈ (0.9, 1) with two set of ICs, (0, 0, 0) (blue plot) and (0.5, 1, −0.2) (red plot); (**b**) two coexisting hidden attractors for *q* = 0.98 (arrow L in Figure 10a) and *q* = 0.9882 (arrow R in Figure 10a) corresponding to two set of ICs, (0, 0, 0) (blue plot) and (0.5, 1, −0.2) (red plot); and (**c**) basin of attraction section x − y of attractors shown in Figure 10b (arrow L), for *q* = 0.98, and an initial condition in the third state variable z = 0. The colors shown in the figure associate with the colors of the attractors given in Figure 10b (arrow L).

**Figure 11:**
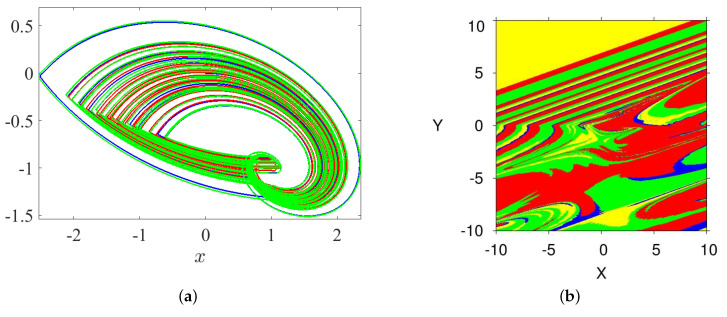
(**a**) Multiple coexisting hidden attractors for three ICs, (0,0,0), (0.5,1,−0.2) and (0.2,−0.2,0.2); and (**b**) basin of attraction section x−y of attractors shown in Figure 11a for q=0.98 according to the IC of the third state variable z=0. The colors of the figure associate with the colors of the attractors given in Figure 11a.

The coexisting attractor of the FoS are deemed as an extraordinary phenomenon. It has recently attracted the attention of several research groups. Actually, the coexisting attractor of a dynamical system relates to its ICs. For the purpose of showing the coexisting attractors of system (5), we plot the bifurcation diagram for q∈(0.9,1) with α=2.8, β=2.8, γ=1, and λ=0.8 in Figure 10a. Two sets of ICs are considered: the first one is (0,0,0), which is represented by the blue plot, and the second one is (0.5,1,−0.2), which is represented by the red plot. The corresponding two plots for the two ICs show that the system exhibits periodic routes to chaos if the commensurate order *q* is increased. For instance, the two coexisting hidden attractors of system (5) are plotted in Figure 10b when q=0.98 (arrow L in Figure 10a) according to the ICs (0,0,0) and (0.5,1,−0.2), which are represented by the blue and red plots, respectively. Two periodic and chaotic hidden attractors coexist when q=0.9882 (arrow R in Figure 10a) according to the ICs (0,0,0) and (0.5,1,−0.2) that are represented by the blue and red plots, respectively. It is noticed that the type of hidden attractors not only depends on the value of *q* but also on the ICs. Actually, the basin of attractions shown in Figure 10c supports these results. In particular, based on this figure, we notice that the ICs represented by the yellow region lead to unbounded orbits, whereas the other ICs represented by the two red and blue regions lead to chaotic attractors. Besides, the system can offer numerous coexisting hidden attractors, as shown in Figure 10c, with three ICs, namely (0,0,0), (0.5,1,−0.2), and (0.2,−0.2,0.2), whereas the corresponding basin of attractions is shown in Figure 11b.

## 4. Incommensurate FoS

**Figure 12:**
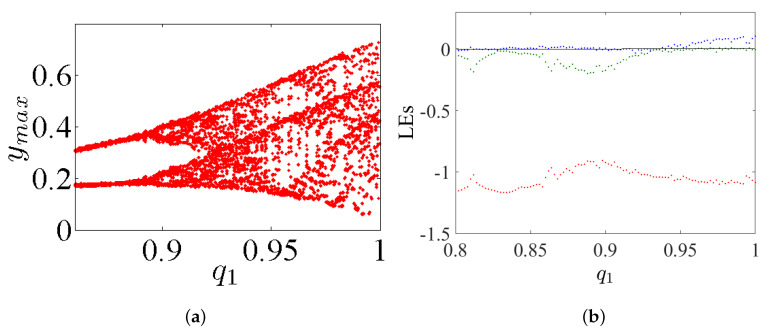
(**a**) The diagram of bifurcation; and (**b**) the LEs of system (5) with incommensurate order by varying $q_{1}\in \left(0.80,\;1\right)$ and fixing $q_{2}=1$ and $q_{3}=1$ with α=2.8, β=2.8, γ=1, λ=0.8 and the IC (0,0,0).

**Figure 13:**
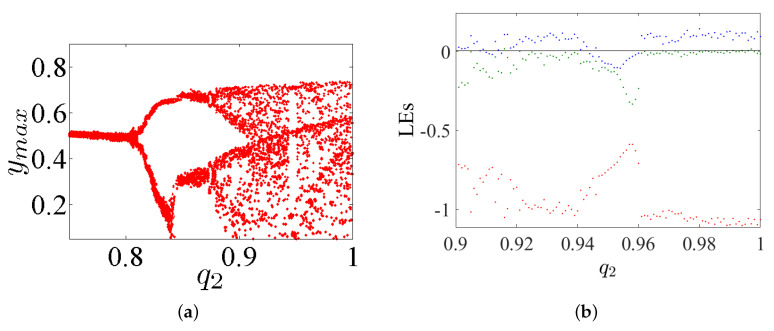
(**a**) The diagram of bifurcation; and (**b**) the LEs of system (5) with incommensurate order by varying $q_{2}\in \left(0.75,\;1\right)$ and fixing $q_{1}=1$ and $q_{3}=1$ with α=2.8, β=2.8, γ=1, λ=0.8 and the IC (0,0,0).

**Figure 14:**
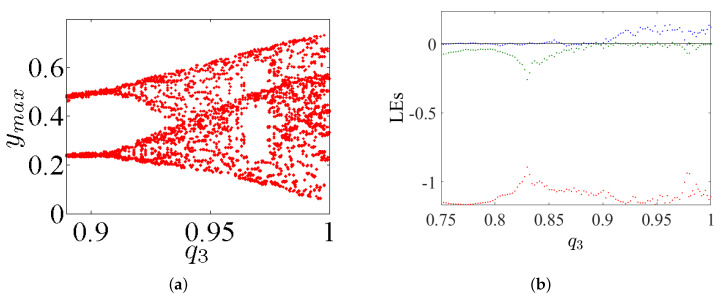
(**a**) The diagram of bifurcation; and (**b**) the LEs of system (5) with incommensurate order by varying $q_{3}\in \left(0.75,\;1\right)$ and fixing $q_{1}=1$ and $q_{2}=1$ with α=2.8, β=2.8, γ=1, λ=0.8 and the IC (0,0,0).

This considers the same dynamics features discussed in the previous section, but this time for incommensurate order. First, we intend to study the dynamic states of this system by varying its incommensurate orders $q_{1}$, $q_{2}$, and $q_{3}$, fixing its parameters α=2.8, β=2.8, γ=1, and λ=0.8, as well as fixing its IC at (0,0,0). The bifurcation diagrams and the LEs of system (5) with its incommensurate order given above are exhibited in Figure 12, Figure 13 and Figure 14, respectively. Actually, these figures display the ranges that illustrate where the system appears in periodic states, quasiperiodic states, and chaos states.

**Figure 15:**
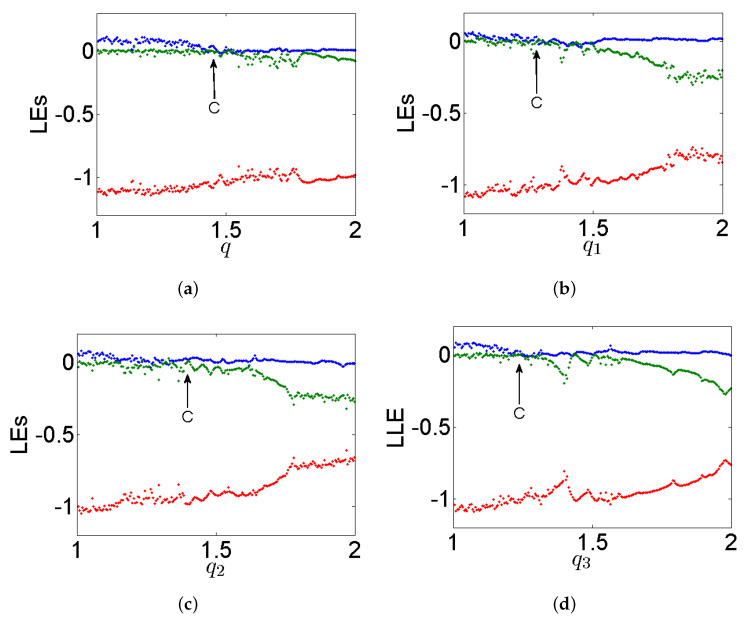
The diagrams of LEs of incommensurate system (5) as function of γ for: (**a**) commensurate order q=0.98; (**b**) incommensurate order $\left[q_{1},q_{2},q_{3}\right]=\left[0.97,1,1\right]$; (**c**) incommensurate order $\left[q_{1},q_{2},q_{3}\right]=\left[1,0.97,1\right]$; and (**d**) incommensurate order $\left[q_{1},q_{2},q_{3}\right]=\left[1,1,0.99\right]$.

With the aim of demonstrating the impact of changing the nature of the fractional-order value on the system’s dynamics, we intend to perform a comparison between the two system’s dynamical states gained from both commensurate and incommensurate cases, in accordance with varying the system’s parameter γ. For this reason, the Lyapunov exponents are calculated and plotted in Figure 15 as a function of parameter γ by selecting the commensurate order as q=0.98 (see Figure 15a) and the incommensurate orders as $\left[q_{1},q_{2},q_{3}\right]=\left[0.97,1,1\right]$, $\left[q_{1},q_{2},q_{3}\right]=\left[1,0.97,1\right]$, and $\left[q_{1},q_{2},q_{3}\right]=\left[1,1,0.99\right]$ (see Figure 15b–d, respectively). It can be seen in these figures that the ranges in which system (5) exhibits chaos are different. As parameter *c* is increased, arrow C in Figure 15 represents the maximum value as possible of *c* where the system generates chaos. In particular, the largest range in which the chaos exists is the range that appears when taking the commensurate order q=0.98, as exhibited in Figure 15a. Besides, the closest range to the chaotic one, which is exhibited from the IoS given in (1), occurs when the incommensurate order $\left[q_{1},q_{2},q_{3}\right]=\left[1,0.97,1\right]$ is taken, see Figure 15c. In general, all these results confirm that the nature of fractional-order value has a key role affecting the dynamics of the FoS.

**Figure 16:**
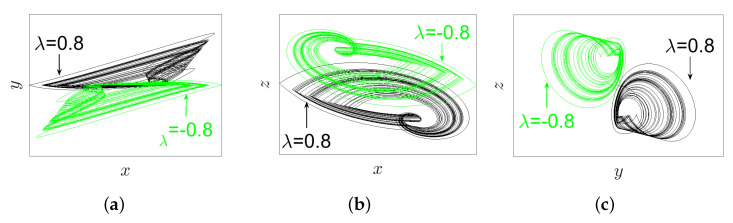
The phase portraits of system (5) with the incommensurate orders $\left[q_{1},q_{2},q_{3}\right]=\left[0.97,1,1\right]$ and α=2.8, β=2.8, and γ=1, in accordance with the IC (0,0,0) and the parameter λ=±0.8 on distinct projections (black plot for λ=0.8, green plot for λ=−0.8): (**a**) xy-plane; (**b**) xz-plane; and (**c**) yz-plane.

For the purpose of exhibiting the inversion property of system (5) with its incommensurate orders, such orders are selected as $\left[q_{1},q_{2},q_{3}\right]=\left[0.97,1,1\right]$; the system’s parameters are set to α=2.8, β=2.8, and γ=1; and the IC is set as (0,0,0). This system has the phase portraits plotted in Figure 16 on distinct projections according to two opposite values of parameter λ=±0.8. In view of such numerical findings, one could conclude that, when the polarity of λ is changed, all system’s signals *x*, *y*, and *z* are consequently changed. This implies that the inversion property still exists if the FoS has incommensurate orders.

**Figure 17:**
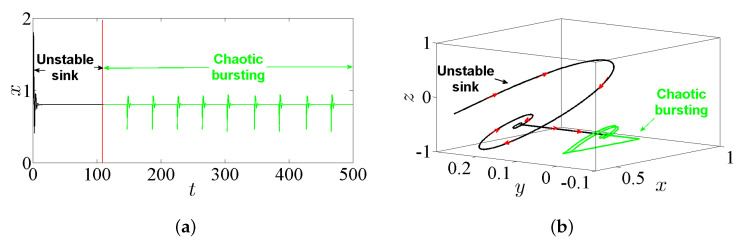
Passing transition behavior by taking the orders $\left[q_{1},q_{2},q_{3}\right]=\left[0.97,1,1\right]$; the parameters α=2.8, β=3.2, γ=1, and λ=0.8; and the IC (1,1,−1): (**a**) the corresponding time series of the state-space variable *x*; and (**b**) the phase portrait in 3D projection.

From another point of view, letting the incommensurate orders be $\left[q_{1},q_{2},q_{3}\right]=\left[0.97,1,1\right]$; the parameters α=2.8, β=3.2, γ=1, and λ=0.8; and the IC (1,1,−1) yields Figure 17, which exhibits the time-domain waveform of the state-space variable *x* (see Figure 17a), and its corresponding phase portrait in 3D projection (see Figure 17b). It can be remarked from these figures that the trajectories of system (5) incur a transition that begins at an unstable sink and ends at a steady chaotic bursting oscillation with the evolution of time. Therefore, a bursting hidden attractor is indeed exhibited for system (5) with its incommensurate orders.

**Figure 18:**
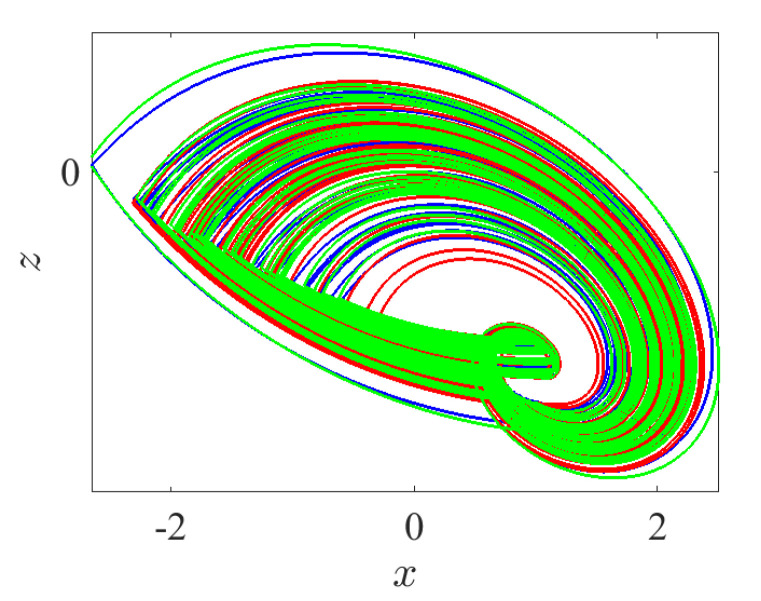
Multiple coexisting hidden attractors according to the three ICs (0,0,0), (0.5,1,−0.2), and (0.2,−0.2,0.2) for $\left[q_{1},q_{2},q_{3}\right]=\left[0.97,1,1\right]$.

In accordance with different incommensurate fractional orders and the three ICs (0,0,0), (0.5,1,−0.2), and (0.2,−0.2,0.2), system (5) can also exhibit multiple coexisting hidden attractors for $\left[q_{1},q_{2},q_{3}\right]=\left[0.97,1,1\right]$, as shown in Figure 18.

## 5. Variable-Boostable Hidden Attractors of Commensurate and Incommensurate FoS

To attain the complete range of the signal’s linear transformations, the offset boosting can be set together with the so-called amplitude control. It appeared that a novel boosting controller, which was introduced by [20], can destroy the symmetry of the variable-boostable system [36,37]. In this section, we introduce three additional controlled constants η, ω, and *ℓ* in accordance with the variables *x*, *y*, and *z*, respectively. The FoS given in (5) then becomes:

(6) $$\left\{\begin{array}{c} D^{q_{1}}x=\alpha sgn\left(y+\omega \right)+\beta \left(z+\ell \right),\\ D^{q_{2}}y=\lambda +\left(z+\ell \right),\\ D^{q_{3}}z=-\gamma \left(x+\eta \right)-\left(z+\ell \right). \end{array}\right.$$

Next, in accordance with α=2.8, β=2.8, γ=1, and λ=0.8, together with the IC (0,0,0), three numerical cases are examined for dealing with the variable-boostable hidden attractors of system (6). Besides, we further select the commensurate and incommensurate fractional-order values as q=0.98 and $\left[q_{1},q_{2},q_{3}\right]=\left[0.98,1,1\right]$, respectively. It should be noted here that all attractors of system (6) are hidden because it has no equilibria irrespective of the system’s parameters, the additional controlled values, and even the initial values.

### 5.1. State 1: A Line of Variable Hidden Attractors

**Figure 19:**
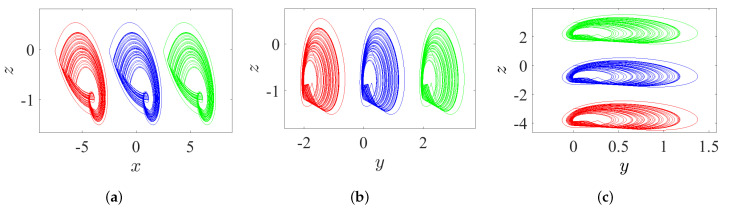
Propagating of the variable one-scroll chaotic hidden attractor on a line corresponding to the IC (0,0,0), and according to γ=1, λ=0.8, α=2.8, β=2.8, and q=0.98: (**a**) *x*-line when η=0 and η=±5; (**b**) *y*-line when ω=0 and ω=±2; and (**c**) *z*-line when ℓ=0 and ℓ=±3.

**Figure 20:**
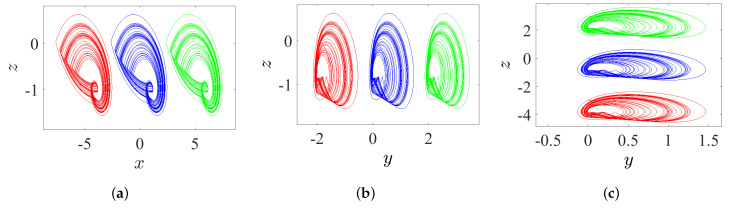
Propagating of the variable one-scroll chaotic hidden attractor on a line corresponding to the IC (0,0,0), and according to γ=1, λ=0.8, α=2.8, β=2.8, and $\left[q_{1},q_{2},q_{3}\right]=\left[0.98,1,1\right]$: (**a**) *x*-line when η=0 and η=±5; (**b**) *y*-line when ω=0 and ω=±2; and (**c**) *z*-line when ℓ=0 and ℓ=±3.

Through controlling each parameter of the offset boosting, a variable hidden attractor can be distributively ordered on a line:

* Once ω=ℓ=0 and η is varied, the variable hidden attractor is diffused on the *x*-axis, as evidenced in Figure 19a for commensurate system and Figure 20a for incommensurate system.
* Once η=ℓ=0 and ω is varied, the variable hidden attractor is diffused on the *y*-axis, as evidenced in Figure 19b for commensurate system and Figure 20b for incommensurate system.
* Once η=ω=0 and *ℓ* is varied, the variable hidden attractor is diffused on the *z*-axis, as evidenced in Figure 19c for commensurate system and Figure 20c for incommensurate system.

### 5.2. State 2: A Lattice of Variable Hidden Attractors

**Figure 21:**
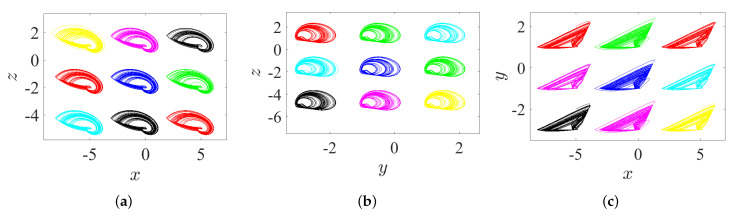
Propagating of the variable one-scroll chaotic hidden attractor on a lattice corresponding to the IC (0,0,0), and according to γ=1, λ=0.8, α=2.8, β=2.8, and q=0.98: (**a**) xz-lattice when (η,ℓ)=(−4,4), (−4,−2), (−4,1), (6,−2), (6,1), (6,4), (1,−2), (1,4), and (1,1); (**b**) yz-lattice when (ω,ℓ)=(3,−2), (3,4), (−1,4), (−1,−2), (−1,1), (1,4), (1,−2), (3,1), and (1,1); and (**c**) xy-lattice when (η,ω)=(6,−1), (6,3), (6,1), (−4,3), (−4,−1), (−4,1), (1,3), (1,−1), and (1,1).

**Figure 22:**
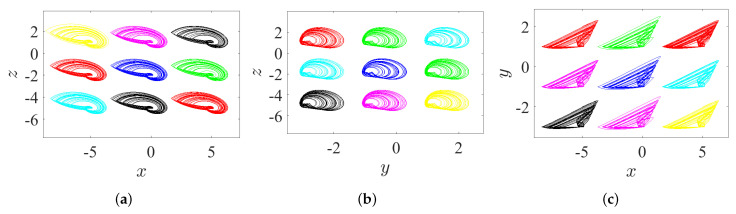
Propagating of the variable one-scroll chaotic hidden attractor on a lattice corresponding to the IC (0,0,0), and according to γ=1, λ=0.8, α=2.8, β=2.8, and $\left[q_{1},q_{2},q_{3}\right]=\left[0.98,1,1\right]$: (**a**) xz-lattice when (η,ℓ)=(−4,4), (−4,−2), (−4,1), (6,−2), (6,4), (6,1), (1,−2), (1,4), and (1,1); (**b**) yz-lattice when (ω,ℓ)=(3,−2), (3,4), (3,1), (−1,4), (−1,−2), (−1,1), (1,4), (1,−2), and (1,1); and (**c**) xy-lattice when (η,ω)=(6,−1), (6,3), (6,1), (−4,3), (−4,−1), (−4,1), (1,3), (1,−1), and (1,1).

To gain a lattice dynamics consisting of variable hidden attractors, one of the controlled parameters should be kept at zero, while the other two should be simultaneously adjusted. However, one can track the following manner for appropriate selection of such combination:

* Once ω=0 and η and *ℓ* are varied, the variable hidden attractors are diffused on the xz-lattice, as demonstrated in Figure 21a for commensurate system and Figure 22a for incommensurate system.
* Once η=0 and ω and *ℓ* are varied, the variable hidden attractors are diffused on the yz-lattice, as demonstrated in Figure 21b for commensurate system and Figure 22b for incommensurate system.
* Once ℓ=0 and η and ω are varied, the variable hidden attractors are diffused on the xy-lattice, as demonstrated in Figure 21c for commensurate system and Figure 22c for incommensurate system.

### 5.3. State 3: A 3D Grid of Variable Hidden Attractors

**Figure 23:**
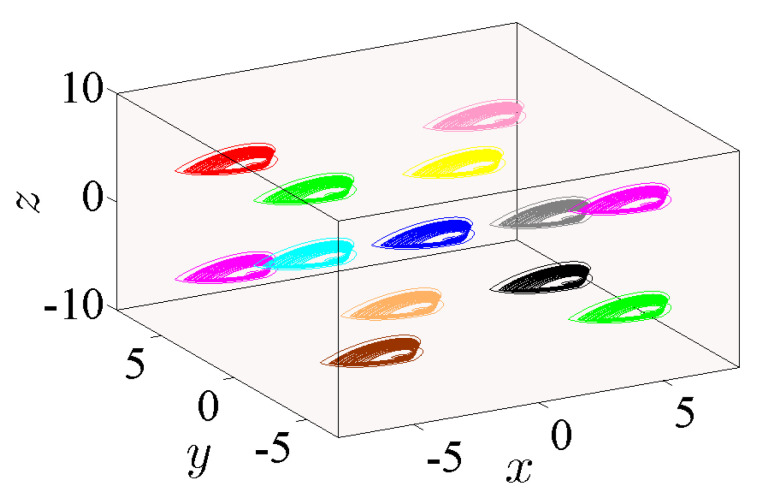
Propagating of the variable one-scroll chaotic hidden attractor on a 3D xyz-grid corresponding to the IC (0,0,0), and according to γ=1, λ=0.8, α=2.8, β=2.8, and q=0.98 for (η,ω,ℓ)=(0,0,0), (−3,−3,−3), (−3,3,−3), (−3,3,3), (3,−3,3), (3,−3,−3), (3,3,3), (−5,−5,−5), (−5,5,−5), (−5,5,5), (5,−5,5), (5,−5,−5), and (5,5,5).

**Figure 24:**
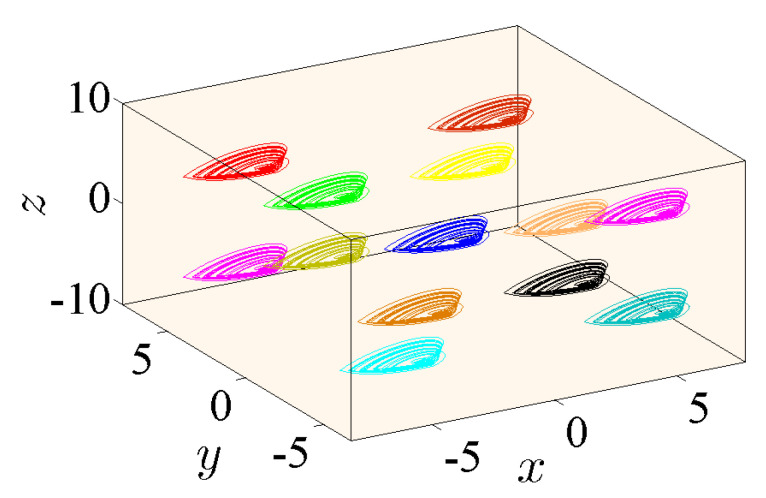
Propagating of the variable one-scroll chaotic hidden attractor on a 3D xyz-grid corresponding to the IC (0,0,0), and according to γ=1, λ=0.8, α=2.8, β=2.8,and $\left[q_{1},q_{2},q_{3}\right]=\left[0.98,1,1\right]$ for (η,ω,ℓ)=(0,0,0), (−3,−3,−3), (−3,3,−3), (−3,3,3), (3,−3,3), (3,−3,−3), (3,3,3), (−5,−5,−5), (−5,5,−5), (−5,5,5), (5,−5,5), (5,−5,−5), and (5,5,5).

**Figure 25:**
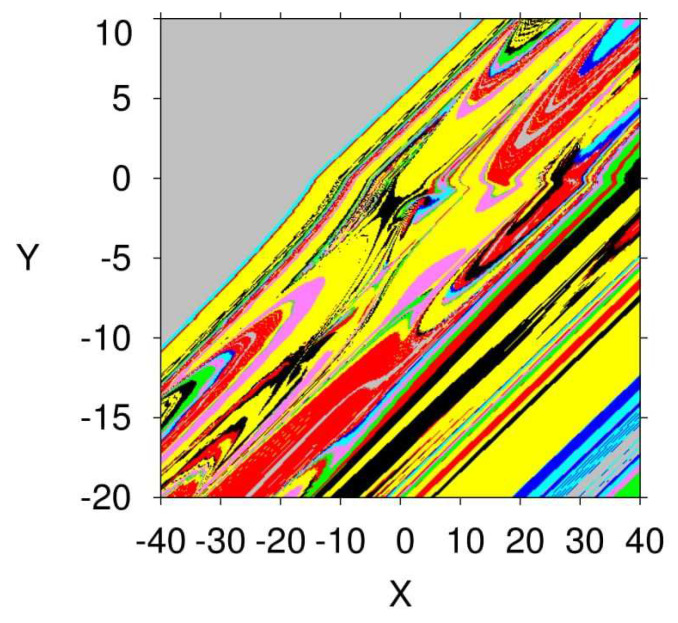
Basin of attraction section x−y of many attractors shown in Figure 24 for $\left[q_{1},q_{2},q_{3}\right]=\left[0.98,1,1\right]$ according to the IC of the third state variable z=0. The colors in this figure associate with the colors of the attractors given in Figure 24.

In this state, all three control parameters η, ω and *ℓ* are simultaneously changed to meet suitable values. The variable hidden attractors are plotted in Figure 23 for commensurate system and Figure 24 for incommensurate system. These attractors are distributively ordered on the xyz-grid. Figure 25 presents the basin of attractions of many attractors that are previously shown in Figure 24 (seven attractors from the grid) for $\left[q_{1},q_{2},q_{3}\right]=\left[0.98,1,1\right]$. In view of this figure, we find that the ICs represented by the gray color lead to unbounded orbits, whereas the other ICs represented by different colors lead to strange attractors. Furthermore, we also find that there is no fixed points in the system for the selected parameters, which implies that the chaotic attractor is hidden.

## 6. Conclusions

A new three-dimensional version of a non-equilibrium chaotic system of fractional-order is established, and its properties and scaling behaviors are explored numerically. Various dynamical behaviors are also revealed for this system, e.g., by examining its dynamic states in accordance with commensurate and incommensurate fractional-order of its derivatives, investigating its dynamic states in accordance with its parameters, knowing whether if it possesses the inversion property, and exploring its hidden chaotic bursting as well as coexisting multiple hidden attractors. It turns out that this fractional-order system has three changeable variables. Besides, the hidden attractors of such system in two cases, the commensurate and incommensurate ones, can be diffused on a 1D line, 2D lattice, and 3D grid, by inserting three additional controlled constants into the system itself.

## Figures and Tables

**Table 1:** Dynamic states of system (5).

*q*	Dynamic State
q∈[0.9000,0.9300)	Period 1
q∈[0.9300,0.9640)	Period 2
q∈[0.9640,0.9720)	Period 4
q∈[0.9720,0.9747)	quasiperiodic
q∈[0.9747,0.9880)	chaos
q∈[0.9880,0.9950)	periodic-route
q∈[0.9950,1.0000]	chaos

## Data Availability

No new data were created or analyzed in this study.
